# Efficiency in Magnocellular Processing: A Common Deficit in Neurodevelopmental Disorders

**DOI:** 10.3389/fnhum.2020.00049

**Published:** 2020-02-26

**Authors:** Alyse Christine Brown, Jessica Lee Peters, Carl Parsons, David Philip Crewther, Sheila Gillard Crewther

**Affiliations:** ^1^School of Psychology and Public Health, La Trobe University, Melbourne, VIC, Australia; ^2^Medical Research Council, Cognition and Brain Sciences Unit, University of Cambridge, Cambridge, United Kingdom; ^3^Port Philip Specialist School, Port Melbourne, VIC, Australia; ^4^Centre for Human Psychopharmacology, Faculty of Health, Arts and Design, Swinburne University of Technology, Hawthorn, VIC, Australia

**Keywords:** magnocellular, flicker fusion, neurodevelopmental disorders, visual processing, autism spectrum disorder, dyslexia, ADHD

## Abstract

Several neurodevelopmental disorders (NDDs) including Developmental Dyslexia (DD), Autism Spectrum Disorder (ASD), but not Attention Deficit Hyperactive Disorder (ADHD), are reported to show deficits in global motion processing. Such behavioral deficits have been linked to a temporal processing deficiency. However, to date, there have been few studies assessing the temporal processing efficiency of the Magnocellular M pathways through temporal modulation. Hence, we measured achromatic flicker fusion thresholds at high and low contrast in nonselective samples of NDDs and neurotypicals (mean age 10, range 7–12 years, *n* = 71) individually, and group matched, for both chronological age and nonverbal intelligence. Autistic tendencies were also measured using the Autism-Spectrum Quotient questionnaire as high AQ scores have previously been associated with the greater physiological amplitude of M-generated nonlinearities. The NDD participants presented with singular or comorbid combinations of DD, ASD, and ADHD. The results showed that ASD and DD, including those with comorbid ADHD, demonstrated significantly lower flicker fusion thresholds (FFTs) than their matched controls. Participants with a singular diagnosis of ADHD did not differ from controls in the FFTs. Overall, the entire NDD plus control populations showed a significant negative correlation between FFT and AQ scores (*r* = −0.269, *p* < 0.02 *n* = 71). In conclusion, this study presents evidence showing that a temporally inefficient M pathway could be the unifying network at fault across the NDDs and particularly in ASD and DD diagnoses, but not in singular diagnosis of ADHD.

## Introduction

The observation that Developmental Dyslexia (DD), Autism Spectrum Disorder (ASD), Intellectual Disability (ID), William’s and Fragile X Syndrome share a deficit in global motion processing led to the formulation of the Dorsal Stream Vulnerability Hypothesis, by Braddick et al. (2003). Since then, many studies have provided further support for the theory (Grinter et al., 2010; Atkinson, 2017) and extended it to include deficits in visuomotor spatial integration for planning actions and attention (Atkinson, 2017). Indeed, epidemiological evidence (Carroll and Owen, 2009; Moskvina et al., 2009) has supported shared symptomology and clustering of neurodevelopmental disorder (NDD) symptoms (DSM-5, American Psychiatric Association, 2013). Implicit in the main hypothesis is the idea that dorsal stream dysfunction in NDDs should be specifically associated with the abnormalities in early magnocellular (M) pathway processing (Braddick et al., 2003; Dakin and Frith, 2005; Laycock et al., 2007). However, to date, there are few studies identifying a specific M defect in NDD, as most tasks have either measured global motion processing using stimuli which are unlikely to stimulate M pathway function alone, or have used stimuli such as gratings that again do not adequately exclude contributions of the parvocellular (P) pathway (Greenaway et al., 2013). In addition, as pointed out by Braddick et al. (2003), the data supporting the hypothesis was largely psychophysical and the term dorsal stream vulnerability was coined to reflect the lack of specificity along the pathways from retina to cortex, as well as avoiding confusion over where the magnocellular pathway (well defined from retina to cortical input), ends. Thus, the aim of this behavioral study was to measure the M pathway function within several NDD populations in a way that could be more effectively related to neurophysiological mechanisms.

The M and P pathways have been shown physiologically to support separate functional information in parallel streams from retina to primary visual cortex (V1; Nassi and Callaway, 2009) though there is some overlap in spatial and temporal responses (Hubel and Wiesel, 1974; Merigan et al., 1981; Nealey and Maunsell, 1994). However, there is evidence in human and primate research that M responses can be isolated physiologically at high temporal frequencies in the retina (Benardete and Kaplan, 1999), LGN (Kaplan, 2004) and V1 (Klistorner et al., 1997; Brown et al., 2018). Lesioning of M layers in monkey LGN shows that the unaffected P neurons can provide a 20 Hz maximum behavioral response (Schiller et al., 1991). This finding is behaviorally supported in adult humans by isolating responses of the P pathway from M pathway *via* the use of isoluminant red/green flicker, which results in a chromatic fusion threshold of around 25 Hz (Wisowaty, 1981) where flicker fusion threshold is defined as the frequency at which modulated light is perceived as constant (Hecht and Shlaer, 1936; Brenton et al., 1989) To this end, behavioral achromatic flicker fusion threshold (FFT; Hecht and Shlaer, 1936; Brenton et al., 1989) has come to be considered theoretically as the most selective behavioral measure of M function (Merigan et al., 1991; Brown et al., 2018). For achromatic flicker fusion, the threshold is reported to lie between 35–64 Hz depending on the temporal contrast i.e., the depth of luminance modulation (Hecht and Shlaer, 1936; de Lange Dzn, 1954). Luminance FFTs have a U-shaped relationship across the lifespan (Tyler, 1989; Kim and Mayer, 1994) peaking around age 16, which is also around the age at which the M pathway is reported to reach adult maturation (Crewther et al., 1999; Klaver et al., 2011).

Recruitment for this study was non-selective in terms of participant diagnoses tested. This resulted in singular and comorbid combinations of ASD, DD and Attention Deficit Hyperactive Disorder (ADHD) diagnoses being included in this study. An ADHD non-comorbid diagnosis has not been associated with visual motion perceptual anomalies, to date, neither have flicker-related studies been reported. However, the inclusion of these ADHD groups will allow us to investigate if comorbid ADHD affects achromatic FFT performance for those NDDs with ASD and/or DD diagnoses. One previous study of coherent motion and form processing in ASD showed that a comorbid ADHD diagnosis did not affect reduced motion sensitivity reported in ASD (Koldewyn et al., 2010).

Reading performance in DD is characterized by a slow reading rate and poor fluency (American Psychiatric Association, 2013). Dyslexia is also generally accompanied by atypical sensory processing in both auditory and visual modalities (Tallal, 1984; Stein and McAnally, 1995; McAnally and Stein, 1996; Stein, 2001). Differences in visible persistence and motion processing sensitivity led to the magnocellular theory of DD (Lovegrove et al., 1980; Stein, 2001) and these discoveries laid the foundation for the dorsal stream hypothesis. Inefficient temporal processing has been demonstrated in adults with dyslexia who were shown to have lower FFT than age-matched controls (Talcott et al., 1998). Consistent with this, the extent of lowered temporal contrast sensitivity in DD becomes greater as a function of temporal frequency (Lovegrove et al., 1980; Martin and Lovegrove, 1987; Mason et al., 1993; Steinman et al., 1997).

While global motion processing was reported to be affected in ASD (Braddick et al., 2003; Dakin and Frith, 2005; Happé and Frith, 2006) these observations have received less support more recently (Kaiser and Shiffrar, 2009; Grinter et al., 2010; Jones et al., 2011; Van der Hallen et al., 2015). In neurotypical adults scoring high in autism traits, lower achromatic FFT has been reported (Thompson et al., 2015). This accords with the high Autism-spectrum Quotient (AQ) physiological literature where high AQ scores are associated with greater amplitude M-generated nonlinearities (Jackson et al., 2013) and are predictive of lower flicker efficiency. Currently, FFTs have not yet been reported in individuals with clinically diagnosed ASD.

Thus, the aim of this behavioral/psychophysical study was to investigate the temporal function of the M pathway in NDD groups *via* measures of achromatic flicker fusion with temporal contrast (depth of luminance modulation) of 5% and 75%. Our first hypothesis was that those with diagnoses of ASD and DD would demonstrate lower FFTs compared to their age and non-verbal IQ matched controls while no group difference would be found for ADHD vs. matched controls. Furthermore, it was hypothesized that ASD and DD participants with a comorbid diagnosis of ADHD would reflect the low FFT results predicted for the singular diagnoses of ASD and DD. Lastly, as visual perception has been reported to be abnormal in neurotypicals high in AQ, it was predicted that across all participants, higher scores in AQ would also relate to lower FFT.

## Materials and Methods

### Participants

Following approval from the Human Research Ethics Committees of La Trobe University and the Victorian Government Department of Education and Early Childhood Development, participants were recruited from mainstream and specialist schools and a school holiday program for children with mild learning delays. Signed consent for the study was obtained from the parent/guardian for all children who participated. This study screened for the presence of epilepsy and excluded these individuals from participating.

Over the course of the data collection period, *n* = 139 participants aged 7–12 years were tested. Out of the 72 typically developing (TD) participants from whom we collected data, 48 were selected to be age and non-verbal intelligence matched controls to the participants with clinical diagnoses. All participants whom we could verify as having a formal clinical NDD diagnose were included in the analyses (*n* = 53). There were six participants excluded because, while they were suspected as having an NDD, they had yet to receive a formal diagnosis. The NDD participants were individually matched to a TD participant within a year of chronological age and within 4 points on non-verbal intelligence, as measured by the Coloured Raven’s Progressive Matrices test (RPM; Raven, 1998), gender was then matched where possible. For demographic information see Table 1. Note that some TD participants were reused as matched controls for participants in different NDD diagnosis groups.

**Table 1:** Different groupings of participants with their age and Raven score-matched controls and Autism-Spectrum Quotient (AQ) score information.

	Clinical					Matched typical controls				
Groups	*N* (M, F)		m	(SD)	Range	*N* (M, F)		m	(SD)	Range
ASD	18 (15, 3)	Year;Month	9;06	(1;03)	7–11	17 (10, 7)	Year;Month	9;06	(1;03)	8–11
		Raven	27.28	(4.43)	19–34		Raven	28.28	(3.94)	21–35
		AQ	88.87	(24.00)	53–128		AQ	44.33	(12.97)	26–65
DD	18 (10, 8)	Year;Month	10;04	(1;03)	7–12	18 (5, 13)	Year;Month	10;06	(1;04)	7–12
		Raven	29.11	(4.12)	20–35		Raven	28.95	(3.74)	22–34
		AQ	52.79	(14.84)	19–80		AQ	44.87	(11.24)	29–63
ADHD	12 (8, 4)	Year;Month	10;05	(1;03)	8–12	12 (7, 5)	Year;Month	9;05	(1;03)	8–12
		Raven	27.17	(4.86)	21–33		Raven	28.08	(4.32)	21–35
		AQ	61.10	(18.60)	16–84		AQ	42.67	(15.22)	16–63
ASD/ADHD	13 (9, 4)	Year;Month	9;06	(2;03)	7–12	13 (8, 5)	Year;Month	9;06	(1;03)	7–12
		Raven	28.31	(4.86)	19–35		Raven	29.00	(4.22)	20–35
		AQ	96.04	(17.52)	74–118		AQ	47.00	(14.07)	16–65
DD/ADHD	6 (4, 2)	Year;Month	9;06	(0;05)	9–10	6 (2, 4)	Year;Month	9;05	(0;02)	9–10
		Raven	27.50	(5.68)	19–34		Raven	27.67	(5.39)	20–34
		AQ	58.20	(16.93)	38–75		AQ	42.00	(18.12)	10–60

### Procedure

Parents/guardians of children with a known or suspected NDD diagnosis were asked to complete an in-house questionnaire that enquired about their child’s clinical diagnostic history. Specifically, they were asked if their child had been formally diagnosed and by what type of professional and that was this a confirmed diagnosis. The questionnaire also asked for parents to specify details of any medication their children were currently taking. Diagnoses of DD were able to be verified experimentally using criteria developed by Cotton et al. (2005). From the ADHD and ADHD+ groups, 29 out of the 31 participants were noted as taking the prescription medication methylphenidate, which is a psychostimulant. The Child AQ (Auyeung et al., 2008) was also completed by all the parent/guardians regardless of the participant group. No control participant scored higher than 76 on the AQ which is the cut off point for clinical ASD in this questionnaire (Auyeung et al., 2008). Testing sessions started with children completing the Coloured RPM, after which they completed two achromatic flicker fusion tasks at high and low contrast. These sessions went for approximately 25 min.

### Flicker Fusion

Two achromatic FFTs were measured at high contrast (75%) and low contrast (5%). FFT was measured using LEDs (A-Bright Industrial Company, Shenzhen, China, part AL-513W3c-003 white) with sinusoidal modulation controlled by the analog output of a VPixx/DATAPixx combination, sampled at 1 kHz, to allow for a smooth variation in temporal frequency. To create a smooth onset/offset to the target flicker and minimize the alerting of change sensitive mechanisms in the visual system, a Gaussian temporal envelope (FWHM = 480 ms) was applied. A ColorCal colorimeter (MkII, Cambridge Research Systems, Rochester, UK) was used to calibrate and linearise the luminance of each light, with an average luminance of 43 cd/m^2^ and the maximum luminance adjusted to 86 cd/m^2^. In our design, four LEDs conveyed their light into separate 6 mm diameter optic fiber light guides. Four holes drilled into a free-standing wooden panel accommodated the light guides in a diamond array.

The task was run in a light controlled, dimly lit room. Participants were seated 60 cm away from the light display and each light guide subtending 1.0° (center-to-center) of visual angle. Participants were informed that one light each trial would flicker for 3 s. At the end of the trial, they were asked to point to the light that they thought had flickered and the experimenter then recorded the participants’ response electronically. The trials were started by the experimenter with a button press once they had ensured that the participant was attending to the display. The onset of the flicker was paired with a high pitch beep to signal the start of a trial and the end of the target flicker was marked with a low pitch beep.

In a four-way alternative, forced-choice design flicker fusion thresholds were established using a Parameter Estimation by Sequential Testing (PEST) Bayesian process, terminated after 32 trials procedure. VPEST is embedded in the VPixx software. The two temporal contrast conditions (5% and 75%) were run separately and were counterbalanced to control for practice effects. To familiarize participants with the task one practice session containing 10 trials was conducted.

### Data Analysis

To test our various hypothesis on group performance, mixed ANOVAs were used to examine each NDD performance on the two FFTs compared to their matched controls. For each participant group, FFT data were checked for outliers two standard deviations away from the mean: none was found. All group data met the requirements of a normal distribution with no significant violation of skewness or kurtosis. Where equal variance was indicated in Levene’s tests, mixed ANOVAs were run. When equal variance was not indicated individual *t*-tests were run and an alpha value 0.025 was used to correct for family-wise error from the two comparisons performed on flicker fusion. To future quantify the differences in group FFT performance we calculated, for each NDD group the percentage of NDD participants whose performance was 1 SD and below their matched control group (see Table 2).

**Table 2:** Percentage of neurodevelopmental disorders (NDDs) who performed 1 SD below their matched controls.

	ASD	Dyslexia	ADHD	ASD/ADHD	Dys/ADHD
FFT 5%	52.94%	43.75%	41.67%	63.63%	66.67%
FFT 75%	68.75%	50%	25%	53.38%	66.67%

There were three instances where the flicker threshold was not properly established after the 32 trials; these data were removed from any further analysis. Lastly, to examine if the AQ score had the predicted negative relationship with FFT one-tailed correlation analysis was chosen which included all participants.

## Results

### Flicker Fusion in Clinical Diagnoses and Matched Controls

#### ASD

**Figure 1:**
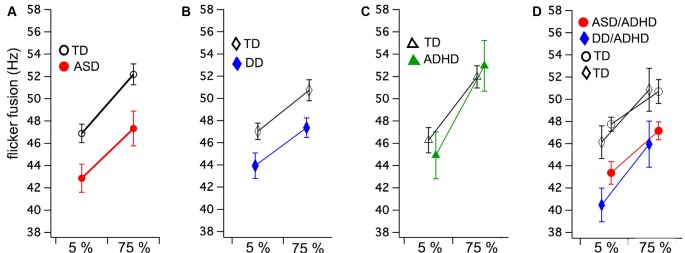
Group means and standard errors for flicker fusion thresholds (FFTs) at low (5%) and high (75%) luminance modulation. Autism spectrum disorder (ASD; **A**) and developmental dyslexia (DD; **B**) both show significantly lower FFTs compared to their matched controls, while no group difference was found in **(C)** attention deficit hyperactive disorder (ADHD). Comorbid ASD/ADHD and DD/ADHD **(D)** with their matched controls (indicated by shared symbols). Separate comparison analyses of these matched groups showed both comorbid groups have significantly lower FFTs than matched controls.

A mixed ANOVA was run comparing two groups (ASD and TD) and flicker fusion (5% and 75% contrast). Within subjects results showed that higher thresholds were obtained in the high contrast flicker condition compared to the low contrast *F*_(1,34)_ = 85.85, *p* < 0.001, *η*^2^ = 0.71, while no interaction between group and flicker condition was found *F*_(1,34)_ = 0.61, *p* < 0.44, *η*^2^ = 0.01. A significant main effect was found between the groups for FFT *F*_(1,34)_ = 7.78, *p* < 0.001, *η*^2^ = 0.19 showing ASD had lower FFT than their matched TD group (Figure 1A).

#### DD

Mixed ANOVA showed that the 75% contrast FFT was higher than the 5% contrast condition *F*_(1,34)_ = 46.30, *p* < 0.001, *η*^2^ = 0.57 while group by condition interaction was found to be insignificant *F*_(1,34)_ = 0.71, *p* < 0.41, *η*^2^ = 0.01. The between groups analyses revealed that those with DD achieved significantly lower FFT than their matched controls *F*_(1,34)_ = 6.06, *p* < 0.02, *η*^2^ = 0.15 (Figure 1B).

#### ADHD

Equal variance according to Levene’s test could not be assumed. As no direction is predicted between the groups, 2 two-tailed independent *t*-tests were run. No group differences between ADHD and their matched TD were found in FFT for either the 5% contrast *t*_(22)_ = −0.57, *p* = 0.58 or 75% contrast conditions *t*_(22)_ = −0.40, *p* = 0.69 (Figure 1C).

#### ASD/ADHD

Mixed ANOVA results showed that higher thresholds were obtained in the high contrast flicker condition compared to the low contrast *F*_(1,22)_ = 26.72, *p* < 0.001, *η*^2^ = 0.54, while no group by condition interaction was found *F*_(1,22)_ = 0.48, *p* = 0.49, *η*^2^ = 0.01. A significant main effect was found between the groups for FFT *F*_(1,22)_ = 11.70, *p* < 0.001, *η*^2^ = 0.35 showing ASD/ADHD had lower FFT than their match TD group (Figure 1D).

#### DD/ADHD

Mixed ANOVA showed that higher thresholds were obtained in the high contrast flicker condition compared to the low contrast *F*_(1,10)_ = 43.47, *p* < 0.001, *η*^2^ = 0.81, while no group by condition interaction was found *F*_(1,10)_ = 0.23, *p* = 0.64. A significant main effect was found between the groups for FFT *F*_(1,10)_ = 4.93, *p* < 0.05, *η*^2^ = 0.33 showing DD/ADHD had lower FFT than their matched TD group (Figure 1D).

### Flicker Fusion in Clinical Group Comparisons

A two by four mixed ANOVA was run comparing FFTs at 5% and 75% contrast with NDD groups ASD, DD, ASD/ADHD, and DD/ADHD. There were no significant differences found between these groups *F*_(3,48)_ = 0.50, *p* = 0.69 on the FFT performance. Further *t*-tests were conducted to establish how ADHD FFT performance compares against all the other NDD participants combined into one group (*n* = 55) and against all controls (*n* = 48) in the study. These groups were comparable for age *F*_(2,112)_ = 0.07, *p* = 0.93 and IQ *F*_(2,112)_ = 0.57, *p* = 0.57. In the 5% contrast condition the ADHD group did not significantly differ in FFT for either the NDDs *t*_(1,63)_ = 1.11, *p* = 0.27 or controls *t*_(1,57)_ = 1.18, *p* = 0.24 however, for the 75% contrast condition there was a clear significant difference between the ADHD and other NDDs *t*_(1,64)_ = 3.74, *p* < 0.001 while no difference was found between ADHD and all controls *t*_(1,57)_ = 1.41, *p* = 0.16.

### 1 SD Below the Mean Analysis

To provide additional information surrounding the FFT profile of each NDD on an individual level, 1 SD below the mean was calculated for each of the matched control groups. Using these data, we counted the number of NDD participants who reached an FFT below their matched controls 1 SD FFT rounded to the nearest two decimal places. This data is reported in Table 2 as percentage of NDD participants performing 1 SD below the mean for each NDD group. Table 2 shows that typically between 41% to 68% of the NDDs performed 1 SD below controls on FFT with the exception of ADHD group for the high contrast flicker condition where only 25% performed 1 SD below their Matched controls.

### AQ Relationship With Flicker Fusion

**Figure 2:**
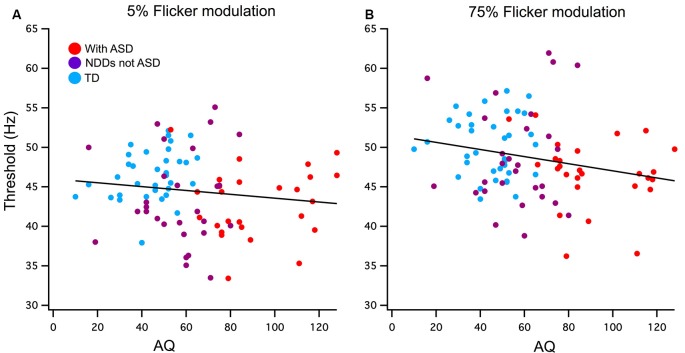
**(A)** Low (5%) and **(B)** high (75%) contrast FFTs correlation with Autism-Spectrum Quotient (AQ) score. Participants have been grouped as typically developing (TD; blue) or neurodevelopmental disorder (NDD) without ASD (purple) and NDD with ASD (red) to highlight the AQ continuation between the groups in this correlation. A significant negative relationship was found between AQ and 75% temporal contrast FFT, however, no significant correlation was found in the 5% temporal contrast condition.

Pearson’s one-tailed correlations between AQ score and the FFTs were run using a list-wise analysis that excludes cases with missing data. Out of the 115 participants analyzed in this study, 20 of those were missing AQ scores due to questionnaires not being completed and returned by parents or guardians. Cook’s distances of 0.5 were used to detect outliers of which there was one in the 75% contrast correlation with AQ, this value was removed. A weak but significant negative relationship was found between AQ and 75% contrast FFT *n* = 90 (*r* = −0.22, *p* < 0.02) and accounts for 5% of the total variance. The low contrast condition did not reach significance *n* = 94 (*r* = −0.13, *p* = 0.21; see Figure 2).

## Discussion

Visual functions such as motion processing, planning for actions and attention are considered to require efficient M pathway processing for efficient processing (Laycock et al., 2007). In this study, our temporal measure of M pathway efficiency—FFT has been observed to be lower in both singular and comorbid diagnosis of ASD and DD with and without ADHD when compared to carefully selected age and IQ matched controls. Furthermore, group differences across the high and low contrast flicker fusion conditions were uniformly observed indicating reasonable stability in these findings. Lastly, the ADHD participants performed similarly in FFT to their matched TDs as was predicted. Interestingly when ADHD was compared to all NDD as one group and all controls it was only in the high contrast condition that ADHD significantly differed from the NDDs while at low contrast such as the range in ADHD FFT values that they did not significantly differ from NDDs or controls. Using 1 SD away from the control group means, no NDD demonstrated more than 2/3 impaired FFT which highlights the heterogeneity and physiological variability underlying clinical diagnoses. Overall, this study presents evidence that a temporally inefficient M pathway could be the common unifying feature across the disorders included in the Dorsal Stream Vulnerability Hypothesis. However, the initial neural locus of temporal inefficiency is still unresolved as the psychophysical techniques used could not distinguish cortical from sub-cortical processing.

In the ASD literature, this study is the first to demonstrate that lower FFT is found in ASD children. This accords with the FFT temporal processing deficit shown in neuro-typical adults rated high in AQ (Thompson et al., 2015). On the other hand, evidence for FFT-based poorer temporal processing in DD has previously been shown in several studies (Talcott et al., 1998; McLean et al., 2011) although a number of other studies more extensive than this have questioned whether only a subset or subtype of DD individuals may experience M-based impairments (Borsting et al., 1996; Williams et al., 2003; Gori et al., 2016; Lawton, 2016). On the other hand, our inclusion of participants with more complex diagnostic profiles has enabled us to show that children comorbid for ADHD/ASD and ADHD/DD perform similarly to children with singular diagnoses of ASD or DD, respectively. This result is consistent with ASD motion coherence performance that was also shown to be unaffected by a comorbid ADHD diagnosis (Koldewyn et al., 2010). Our findings also present an argument, for the potentially unnecessary exclusion of participants with comorbid ADHD from visual perception research. This is particularly true if sustained attention is not required, as was the case in our task that only required 3 s of focus for each trial. Indeed, including more complex diagnostic profiles has the benefit of increasing sample sizes and the generalizability of the study’s results in a research area that often suffers from being underpowered (Loth et al., 2017). Interestingly the low contrast condition, it is apparent that some ADHD participants with high AQ scores do show evidence for slower temporal processing.

Of the groups of NDDs associated with the dorsal stream theory, ASD stands out with regard to their unique local perception profile as Grinter et al. (2010) have highlighted, atypical visual processing in this population is the most likely to extend beyond the dorsal stream. Interestingly, Van der Hallen et al. (2015) theorized that the perceptual differences in ASD resided in the speed with which global order is perceived, suggesting a change to the temporal local/global balance.

The weak negative relationship that was also found between AQ scores and FFTs in the high 75% contrast flicker fusion condition is the first to show that AQ can predict some of the variations in a task chosen to test M-type function and visual processing across a sample consisting of multiple clinical disorders and neurotypicals.

The chief limitation of this study is the lack of an ability to relate the magnocellular pathway processing in NDDs to neural sites. Such an extension would require brain imaging with rapid temporal resolution—perhaps best performed using MEG. Furthermore, extension of this research to an investigation of P temporal processing using red/green isoluminant flicker would be useful to establish the generality of such a temporal processing deficit.

In conclusion, this study presents convincing evidence for M inefficiency in ASD and DD and suggests the continued investigation of temporal M processing in other NDDs such as Rett Syndrome and Williams Syndrome, included in the original Dorsal Stream Hypothesis. Although inefficient temporal M processing is not going to provide a singular limiting constraint accounting for all the visual abnormalities in NDDs, it is a major contributor.

## Data Availability Statement

The datasets generated for this study are available on request to the corresponding author.

## Ethics Statement

The studies involving human participants were reviewed and approved by/from Human Research Ethics Committees of La Trobe University Victorian Government Department of Education and Early Childhood Development. Written informed consent to participate in this study was provided by the participants’ legal guardian/next of kin.

## Author Contributions

AB was the primary contributor to this study and was involved in the design, research theory, data collection, analysis and write up. JP was a PhD student who helped with the data collection, clinical aspects of the study and drafting. CP helped with data collection, recruitment and clinical aspects of the study. DC and SC co-supervised this study and were involved in the design and the development of the theory and helped with the write up of the manuscript.

## Conflict of Interest

The authors declare that the research was conducted in the absence of any commercial or financial relationships that could be construed as a potential conflict of interest.
